# Removal of radiation-related tattoos among breast cancer survivors using a 20-nanosecond Q-switched Ruby laser

**DOI:** 10.1007/s10103-026-04798-4

**Published:** 2026-03-18

**Authors:** Eran Sharon, Gaia Harris Rimon, Noam Weiner, Mohammad Najjar, Shiri Daniel Mimouni, Lior Har-Shai, Moshe Lapidoth, Assi Levi, Yehonatan Noyman

**Affiliations:** 1 Breast Surgery Unit, Department of Surgery, Rabin Medical Center, Petah Tikva, Israel; 2 Division of Dermatology, Rabin Medical Center, Petah Tikva, Israel; 3 The Department of Reconstructive Plastic Surgery and Burns, Rabin Medical Center, Petah Tikva, Israel; 4 Gray Faculty of Medical and Health Sciences, Tel Aviv University, Tel Aviv, Israel

**Keywords:** Q-Switched ruby laser, QSRL, Laser, Tattoo, Breast cancer, Radiotherapy

## Abstract

**Background:**

Permanent dark ink tattoos for radiotherapy (radiation-related tattoos) play a critical role in breast cancer management by ensuring accurate field localization and consistent patient positioning. While effective, these markings persist permanently and may negatively impact body image and quality of life. Laser tattoo removal using selective photothermolysis is the standard of care for tattoo removal, with the 20-nanosecond Q-switched ruby laser (QSRL) particularly suited for targeting dark dermal pigment. We evaluated the safety and efficacy of QSRL for the removal of radiation-related tattoos in breast cancer survivors.

**Methods:**

A prospective, single-center interventional study of female breast cancer survivors with radiation-related tattoos. Eligible participants underwent up to 6 QSRL treatment sessions. Outcomes were assessed by 2 independent dermatologists through blinded evaluation of pre- and post-treatment photographs using the Global Aesthetic Improvement Scale (GAIS), patient satisfaction scores, and body-image questionnaires. Adverse effects were documented as well.

**Results:**

Seven patients were included with a total of 23 tattoos. Significant improvement or complete resolution (average GAIS ≥ 3) was noted in 91% of treated tattoos, with strong inter-rater agreement (87%). Treatment was well-tolerated, with mild-to-moderate, short-lived pain during the procedure and minimal downtime. Patient satisfaction was high and no adverse events were reported.

**Conclusion:**

QSRL is a safe, effective, and well-tolerated modality for removing radiation-related tattoos in breast cancer survivors and may represent a valuable component of comprehensive post-treatment care for eligible patients.

**Supplementary Information:**

The online version contains supplementary material available at 10.1007/s10103-026-04798-4.

## Introduction

 Iatrogenic tattoos play an essential role in radiotherapy for breast cancer, facilitating precise field localization and consistent patient positioning throughout treatment [1].

Temporary marker pens, henna, and permanent dark ink tattoo are commonly used to create 2–3-mm skin markings for radiotherapy alignment (radiation-related tattoos), with permanent tattoos being the most widely adopted method globally, accepted by nearly all radiotherapy departments. This preference reflects their durability, reproducibility, and cost-effectiveness compared with temporary methods that fade within days to weeks [1, 2]. Though highly effective, the permanent nature of ink tattoos has been associated with psychological distress and reduced quality of life. Following radiotherapy for breast cancer, 63% of participants reported negative impact of the tattoos, including feelings of aversion [3, 4].

Selective photothermolysis allows targeted destruction of specific chromophores through precisely timed laser pulses at wavelengths matched to the chromophore’s absorption spectrum. It is regarded as the treatment of choice for tattoo removal and is also used for scars and pigmentary lesions [5–9]. The 694-nm Q-switched ruby laser (QSRL) is being successfully used for the treatment of cutaneous pigmentary lesions and dark tattoos [10], making it a promising option for radiation-related tattoos, with potential benefits for quality of life.

To date, the evidence on laser-assisted removal of radiation-related tattoos is limited. One study compared the 1,064-nm Q-Switched Neodymium-doped Yttrium Aluminum Garnet (Nd:YAG) with punch biopsy excision. Both methods were similarly effective, but excision was associated with more frequent and pronounced adverse events of hypopigmentation and scarring, making laser treatment the preferred option among patients [11]. A physician survey further emphasized the need for validated clinical data [12]. Although QSRL has the highest melanin absorption coefficient compared with other available lasers, no current evidence supports its use for the removal of radiation-related tattoos.

Given the established effectiveness of QSRL in tattoo removal, we hypothesized that it would be effective for the removal of radiation-related tattoos. Accordingly, this study aimed to evaluate the safety and efficacy of QSRL for this indication.

## Methods

### Study design and setting

A prospective, single-center interventional study was conducted among breast cancer survivors treated with QSRL for the removal of radiation-related tattoos. Treatments took place between February 2023 and May 2024 at a tertiary medical-center dermatology laser unit.

The study was approved by the institutional Ethics Committee (RMC 0761-22).

## Patients

All participants were referred from the Breast Cancer Unit.

Eligibility criteria included female patients aged ≥ 18 years with primary breast cancer, no evidence of distant metastasis, completion of oncologic treatment, and persistent radiation-related tattoos, no longer required for radiation localization.

Exclusion criteria included faint pigmentation, Fitzpatrick skin types V–VI, contraindications to laser therapy, pregnancy, or lactation (Appendix 1).

## Treatment protocol

Before the first treatment, patients received a comprehensive explanation regarding the objectives, protocol, potential adverse events, and the option of watchful waiting. Patients provided a written informed consent and were photographed.

As no validated pigmentation-specific tool exists for this population, patients completed an author-designed body-image questionnaire comprising 7 items on a 1–5 scale (1 – strongly disagree, 5 – strongly agree), with equal weight assigned to each item (Appendix 2).

Topical lidocaine-prilocaine cream was applied according to patient’s preference.

Treatments were performed by one of two board-certified dermatologists (A.L. or Y.N.), using QSRL (Sinon, Alma Lasers, Caesarea, Israel) with a 20-ns pulse duration. Each session consisted of a single pass, with parameters set at a 4-mm spot size and a fluence of 4–6.5 J/cm². Fluence was adjusted in order to achieve the clinical endpoint of immediate slight blanching of the tattoo color. Immediately following treatment, pain was assessed using the Visual Analogue Scale (VAS) ranging from 0 to 10 (0 – no pain, 10 – worst possible pain; Appendix 3). Post-treatment care included moisturizer application. Patients received written instructions to avoid sun exposure for 2 weeks after each treatment and for 2 weeks before the next, and to apply moisturizer as needed. Downtime was assessed at the subsequent visit using a dedicated questionnaire (Appendix 4).

Up to 6 treatment sessions were conducted at 4–8-week intervals to allow for adequate healing and informed assessment of the need for further treatment based on pigmentation status.

Treatment was discontinued upon complete resolution, reaching a maximum of 6 sessions, or at patient request. A follow-up visit 4–8 weeks after the final session included repeated photography, patient satisfaction assessment using a 5-point scale (1 – completely dissatisfied, 5 – very satisfied; Appendix 5), and repeated body-image questionnaire.

## Outcome measures

Pigmentation improvement was independently evaluated by 2 dermatologists not involved in the treatment using pre- and post-treatment photographs presented in a randomized order, with each tattoo assessed independently, rather than as part of a sequential review of a given patient’s tattoos.

Evaluators were first asked to identify which image was taken before treatment and which after, and then to rate the degree of improvement using the Global Aesthetic Improvement Scale (GAIS), ranging from 0 (no improvement) to 4 (complete resolution).

Primary outcomes included the degree of pigmentation improvement, calculated as the mean rating of the 2 evaluators, patient satisfaction, and changes in body image perception.

Secondary outcomes included the frequency and severity of adverse events and the total number of treatment sessions required.

### Statistical analysis

Participants’ demographic and clinical data were collected in de-identified format and summarized using descriptive statistics. Categorical variables are presented as counts (n), and continuous variables as means and ranges.

## Results

Nine female patients were enrolled. Seven were included in the analysis (all Fitzpatrick skin types I–II), and two withdrew after 1–2 treatment sessions due to unwillingness to adhere to the study schedule.

The mean age at first treatment was 51 years (range 45–67 years), and the mean time since tattoo placement was 5.1 years (range 1–14 years).

A total of 23 radiation-related tattoos (2–4 per patient) were treated, requiring a mean of 4 sessions (range 2–6), determined by clinical response.

Both independent evaluators correctly identified all pre- and post-treatment images. Inter-rater agreement for clinically meaningful improvement (GAIS ≥ 3) was 87%.

**Fig. 1:**
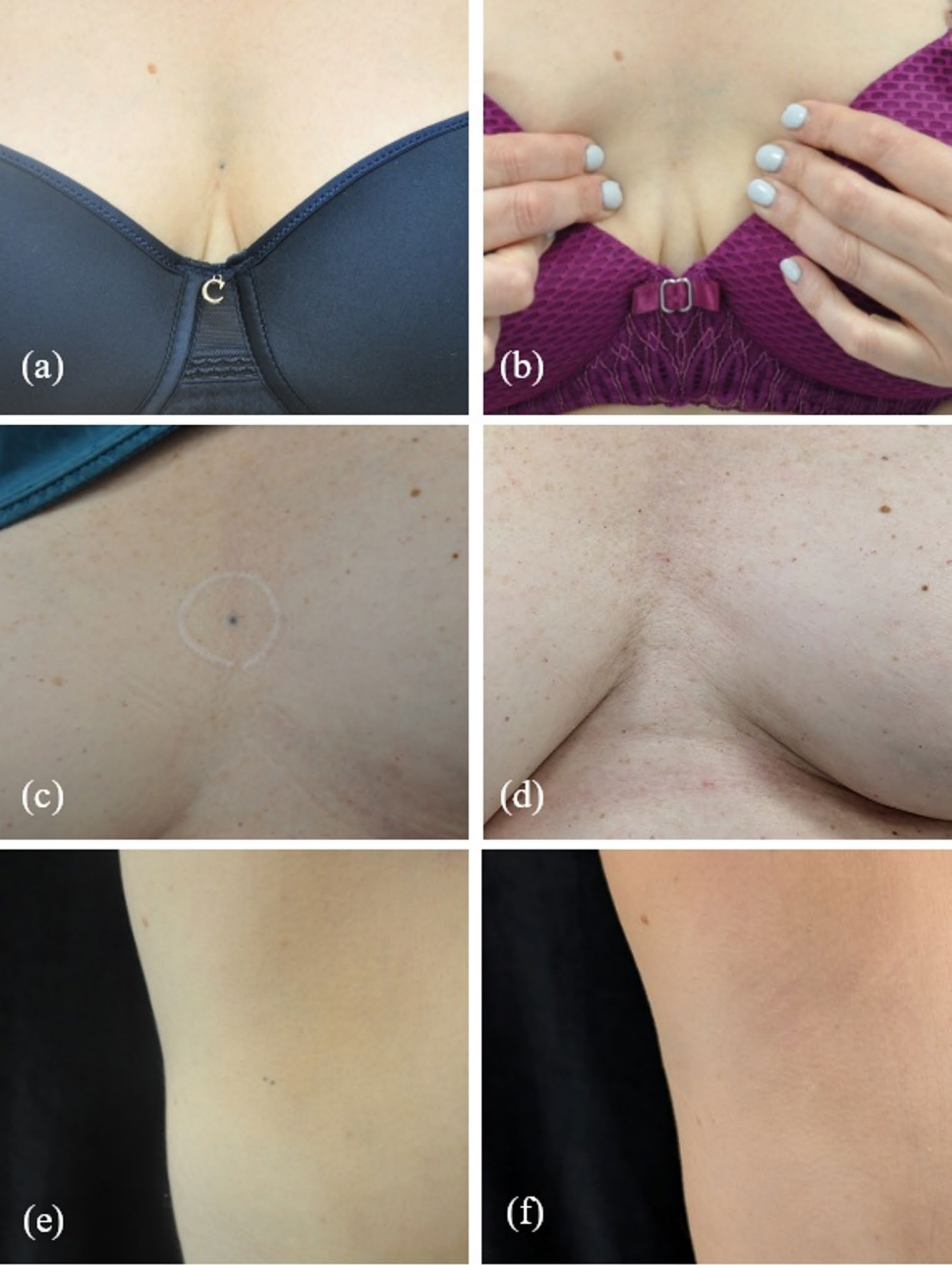
Representative radiation-related tattoos before and after QSRL treatment. Pre-treatment images are shown on the left and post-treatment images on the right. Patient #2 (**a**, **b**) and patient #3 (**c**, **d**) underwent 5 sessions each; Patient #4 (**e**, **f**) underwent 2 sessions

Of 23 tattoos, 21 (91%) achieved significant improvement or complete resolution. Three representative cases are shown in Fig. 1.

No correlation was observed between clinical response and time since tattoo placement, performing medical facility, or anatomical location.

All patients resumed their daily routines immediately. Treatment-related pain was mild-to-moderate (mean VAS 4.6/10), short-lived, and consistent across sessions. From the second treatment onward, most patients declined the use of topical anesthesia.

Downtime consisted of mild transient local erythema, tenderness, desquamation, or swelling, typically resolving within hours to days. One patient experienced prolonged erythema and desquamation lasting 2 weeks. No adverse events were reported.

All patients reported maximal satisfaction (5/5).

No significant change in body-image questionnaire scores was observed.

Demographic and clinical characteristics of the patients are summarized in Table 1.

**Table 1:** Demographic and clinical data of breast cancer survivors treated with QSRL for removal of permanent dark ink tattoos used for radiotherapy field marking

Patient	Age (y)	Total tattoos treated (*n*)	Session count (*n*)	Tattoos with score = 3^a^ (*n*)	Tattoos with score = 4^b^ (*n*)	Satisfaction^c^
1	67	3	5–6	2	0	5
2	49	3	5	1	2	5
3	61	4	4–5	3	1	5
4	48	2	2	0	2	5
5	45	4	4–5	1	3	5
6	45	3	2–4	0	2	5
7	46	4	3–4	1	3	5

Abbreviations: y, years; n, number

^a^Score=3: Mean GAIS of 3 or 3.5

^b^Score=4: Mean GAIS of 4

^c^Graded on a 5-point scale (1 – completely dissatisfied, 5 – very satisfied)

### Discussion

 In this prospective interventional study, we demonstrated that QSRL treatment is both safe and effective for the removal of radiation-related tattoos in breast cancer survivors. Significant improvement or complete resolution was achieved in 91% of treated tattoos, with high inter-rater agreement and excellent patient satisfaction. No adverse events were reported, and all patients resumed their daily activities immediately.

 Radiation-related tattoos are routinely used in other malignancies, including prostate, colorectal, and gynecologic cancers [1]. While their importance during radiotherapy is undisputed, these markings persist long after treatment completion. For many patients, they serve as a constant visual reminder of the disease and may carry a disproportionate psychological burden. Notably, several patients in our study had other, larger, and more clinically apparent lesions, such as seborrheic keratoses, that did not elicit emotional distress comparable to that associated with the small radiation-related tattoos. This observation supports the notion that the symbolic significance of these markings often outweighs their physical attributes in shaping patients’ perceptions. Such permanent markings can negatively affect body image, self-esteem, and quality of life, and may interfere with the psychological transition from active treatment to survivorship [1, 13, 14].

 In response to a growing recognition of the emotional burden these markings can impose, alternative tattooing methods [15] and nonpermanent localization strategies are being explored, including surface-guided radiation therapy, temporary skin markers, and ultraviolet-ink tattoos [1, 16, 17]. However, such methods are not yet universally available, particularly in resource-limited settings [2, 18]. Therefore, there remains a considerable need for safe, effective post-treatment options for patients wishing to remove radiation-related tattoos.

Owing to the established role of lasers in tattoo removal, we used a laser-based approach. Specifically, QSRL was selected because the ruby laser’s 694-nm wavelength has the highest melanin absorption among the available lasers, making it especially attractive for this indication [19–22]. Prior work using a 1,064-nm Q-switched Nd:YAG laser demonstrated that laser treatment is an effective and better-tolerated alternative to punch-biopsy excision for radiation-related tattoo removal, supporting the use of laser-based modalities for this indication [11].

Our study advances the field by being the first to evaluate the pigment-selective 694-nm QSRL for this indication, leveraging its highest melanin absorption coefficient [10]. We also introduce a more rigorous, patient-centered evaluation framework – randomized, blinded, tattoo-level GAIS rating by two dermatologists, systematic downtime capture, and satisfaction metrics – and analyze a larger series (23 markings vs. 10), enabling more established and clinically relevant insights. Although a direct head-to-head comparison was not conducted, our patient satisfaction scores appear favorable, with all patients reporting a satisfaction of 5/5. This compares with the previously reported median satisfaction of 9/10 (interquartile range, 5.75–10) for the 1,064-nm laser approach [11].

Treatment was well-tolerated with moderate, short-lived pain levels and no adverse events. Downtime was minimal, lasting hours to days. These observations are consistent with existing literature describing the favorable safety profile of QSRL when used correctly [19, 23].

As expected, most tattoos showed significant improvement to complete resolution over several treatment sessions. However, the number of required sessions varied. This variability was not explained by skin type, time since tattoo placement, anatomical location, or medical facility at which the tattoo was applied. It may reflect natural individual variation, which could be less apparent in larger cohorts. These findings underscore the heterogeneity of clinical responses and highlight the need for individualized treatment planning to optimize outcomes. Further studies are warranted to explore this variability.

This study has several limitations. First, the small sample size limits the generalizability of the findings and precludes robust subgroup analysis. Second, the lack of a control group (e.g., alternative laser modalities) restricts comparative evaluation. Third, one of the outcome measures was self-reported satisfaction and is therefore inherently subjective; however, it correlated with the objective, blinded assessment conducted by two independent dermatologists. Forth, only patients with Fitzpatrick skin types I–II were enrolled, whereas types III–IV were not represented in the study group and types V–VI were excluded. The potential limitations of QSRL in darker skin types must therefore be acknowledged, particularly given the increased risk of hypopigmentation. Although a relatively low incidence of hypopigmentation was reported in this population [24, 25], further studies are required to evaluate this technique in individuals with darker skin tones.

In conclusion, our findings support QSRL as a safe and effective option for removing radiation-related tattoos in breast cancer survivors. As with many aspects of survivorship, the elimination of cancer does not necessarily mark the end of a patient’s therapeutic trajectory. Psychosocial rehabilitation, including support groups and body image counselling, are already recognized components of holistic post-cancer care.

Given the limited published research, this study provides important early evidence and is the first to specifically evaluate QSRL – the preferred laser for dark tattoos – in this context. Laser removal of radiation-related tattoos may represent a meaningful component of survivorship care for selected patients.

## Supplementary Information

Below is the link to the electronic supplementary material.

Supplementary Material 1

## Data Availability

The data that support the findings of this study are available from the corresponding author [YN] upon reasonable request.
