# Supplementary material for: Removal of radiation-related tattoos among breast cancer survivors using a 20-nanosecond Q-switched Ruby laser
Source: Lasers Med Sci. 2026 Mar 18;41(1):58. doi: 10.1007/s10103-026-04798-4 (PMC12999598; doi:10.1007/s10103-026-04798-4)
Supplement: Supplementary file 1 — Supplementary Material 1 [file 10103_2026_4798_MOESM1_ESM.docx]

Appendix 1: Eligibility assessment

Inclusion Criteria – **a** **negative answer to any** of the following questions excludes participation in the study

| ☐ Yes ☐ No | Diagnosis of non-metastatic breast cancer |
| --- | --- |
| ☐ Yes ☐ No | Age above 18 years |
| ☐ Yes ☐ No | Completion of cancer treatment with no evidence of disease (NED) |
| ☐ Yes ☐ No | Presence of cutaneous dyspigmentation due to radiation field marking |
| ☐ Yes ☐ No | Patient is willing and able to participate in the study according to study requirements and scheduled visits |
| ☐ Yes ☐ No | Patient has signed informed consent |

Exclusion Criteria – **a** **positive answer to any** of the following questions excludes participation in the study

| **General** | |
| --- | --- |
| ☐ Yes ☐ No | Mild dyspigmentation |
| ☐ Yes ☐ No | Fitzpatrick skin type 5-6 |
| ☐ Yes ☐ No | Photosensitivity to visible light |
| ☐ Yes ☐ No | Immunosuppression or use of immunosuppressive medications |
| ☐ Yes ☐ No | Undergoing diagnosis or active treatment for cancer |
| ☐ Yes ☐ No | Surgery or other treatment in the area of dyspigmentation within the past 6 months |
| ☐ Yes ☐ No | Concurrent participation in another clinical trial or planned participation in another study during the trial period |

| **Risk of scarring** | |
| --- | --- |
| ☐ Yes ☐ No | **Tendency to scarring, hypo- or hyperpigmentation** |
| ☐ Yes ☐ No | **Oral retinoid therapy within the past 6 months, or treatment with psoralen** |
| **Skin** | |
| ☐ Yes ☐ No | **History of skin diseases with risk of Koebnerization –** psoriasis, lichen planus, vitiligo |
| ☐ Yes ☐ No | **Active infection or inflammation in the treatment area** |
| ☐ Yes ☐ No | **History of herpes simplex infection in the treatment area** |
| ☐ Yes ☐ No | **Overlapping lesion on the dyspigmented area (collision)** |
| ☐ Yes ☐ No | **Pregnancy or breastfeeding** |
| ☐ Yes ☐ No | **Recent tanning and/or phototherapy in the treatment area within the past 2 weeks** |

Appendix 2: Body image questionnaire

Please read the following items carefully and circle the answer that best reflects how you felt about your body during the past week.

|  | Strongly disagree | Disagree | Neutral | Agree | Strongly agree |
| --- | --- | --- | --- | --- | --- |
|  | 1 | 2 | 3 | 4 | 5 |
| I try to hide my body |  |  |  |  |  |
| I am satisfied with my appearance |  |  |  |  |  |
| I feel less feminine as a result of the cancer |  |  |  |  |  |
| I feel that my body has let me down |  |  |  |  |  |
| I feel that I need to hide parts of my body |  |  |  |  |  |
| The appearance of my breast may be disturbing to others |  |  |  |  |  |
| I avoid intimate situations |  |  |  |  |  |

Appendix 3: Pain assessment questionnaire

Circle the number that best represents the intensity of the pain you experienced during the treatment


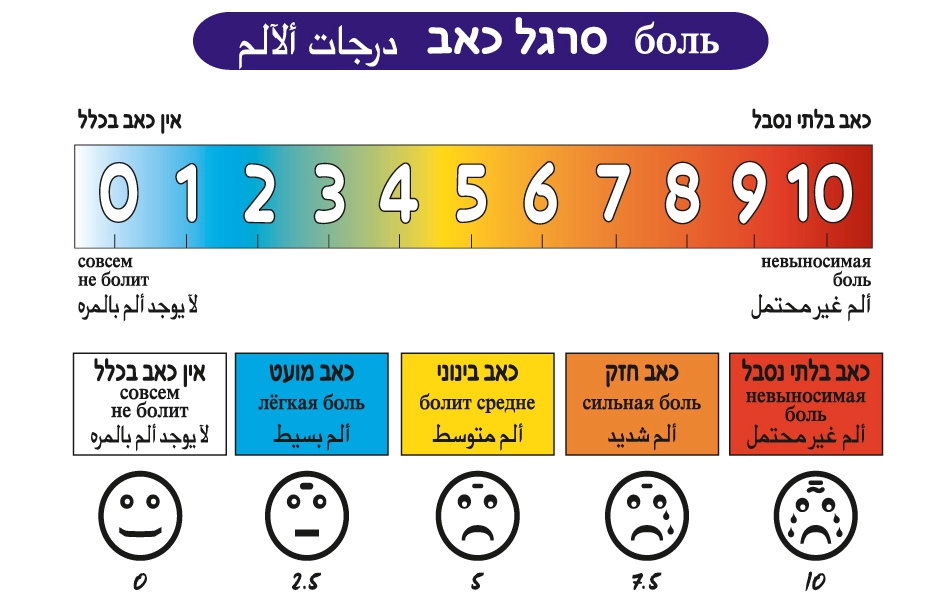


**No pain**

**Worst possible pain**


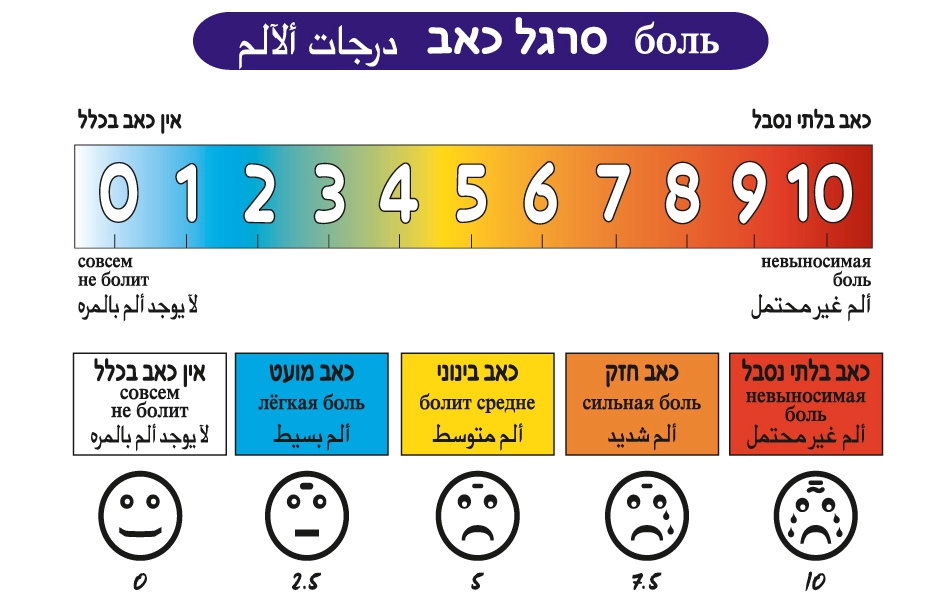


Appendix 4: Post-treatment downtime assessment form

**Symptom Duration**

Local tenderness ______ hours/days

Redness ______ hours/days

Swelling ______ hours/days

Peeling/desquamation ______ hours/days

Itching ______ hours/days

**Limitations Time**

Return to social activities ______ hours/days

Return to work ______ hours/days

Appendix 5: Satisfaction with the treatment questionnaire

Please circle the number that best reflects your satisfaction with the treatment:

1 – Not satisfied at all

2 – Dissatisfied

3 – Neutral

4 – Satisfied

5 – Very satisfied
